# In and out: factors influencing two decades of straying and homing by Pacific salmon within the Columbia River basin

**DOI:** 10.1098/rsos.250447

**Published:** 2025-08-20

**Authors:** Peter A. H. Westley, Andrew H. Dittman, Benjamin W. Nelson, Morgan H. Bond, Molly Payne, Thomas P. Quinn

**Affiliations:** ^1^ College of Fisheries and Ocean Sciences, University of Alaska Fairbanks, Fairbanks, AK, USA; ^2^ School of Aquatic and Fishery Sciences, University of Washington, Seattle, WA, USA; ^3^ NOAA Fisheries Northwest Fisheries Science Center, Seattle, WA, USA; ^4^ Personal consultant, USA

**Keywords:** temperature, dispersal, philopatry, metapopulation dynamics, salmonid fishes

## Abstract

In addition to patch size and spatial isolation, habitat quality can also influence attractiveness of patches to dispersing individuals. Using mark recapture data from 19 populations of Chinook salmon (*Oncorhynchus tshawytscha*) across 23 years in the Columbia River basin, USA, we estimated rates of emigration and immigration among sites, yielding 358 site-year comparisons of donor and recipient straying, representing over 1.5 million individual tag recoveries. The factors associated with emigration and immigration were quantified with a Bayesian zero-inflated beta regression model. We found lower rates of straying from larger basins than from smaller basins and that donor and recipient straying decreased in years when relatively high numbers of salmon from a given river returned compared with years when fewer returned. Additionally, water temperature was positively associated with both immigration into and emigration out of hatcheries, increasing by approximately 0.5% for every degree of warming, suggesting consequences for both donor and recipient populations. This work suggests that dispersal rates may increase in a warming world, which in turn has implication for the flow of both individuals and, potentially, beneficial or deleterious genes.

## 1. Introduction

The metapopulation concept is a unifying ecological framework for studying populations in fragmented landscapes [1–3]. Although the term metapopulation has been applied with several meanings [4], it most appropriately describes a set of local populations that: (i) occupy discrete habitat patches, (ii) exist in a dynamic balance between extinction and colonization and (iii) are connected by dispersal [5]. The metapopulation concept has been examined extensively with respect to the roles of patch size and isolation [6–8], yet patch *quality* also influences metapopulation dynamics [9]. Indeed, both theoretical (e.g. [10]) and empirical assessments [11,12] indicate that habitat quality influences metapopulation processes and results in a patchwork of site attractiveness [13,14]. Habitat quality appears to be a particularly important aspect of metapopulation dynamics in taxa that can ‘choose’ which sites to occupy [15,16].

Much of our knowledge of metapopulation dynamics is gleaned from terrestrial organisms, but there are also excellent aquatic model systems. For example, Pacific (*Oncorhynchus* spp.), Atlantic salmon (*Salmo salar*) and their relatives are fishes with profound cultural, ecological and economic importance to human societies, exhibiting archetypical characteristics of metapopulations [17]. Salmon breed in discrete ‘patches’ of suitable stream habitat that vary in quality over time and space. Natal philopatry of breeding groups, termed ‘homing’, leads to reproductive isolation that is balanced through dispersal, termed ‘straying’ [17–19]. Straying and homing are fundamental aspects of salmon life history as they shape the ecology and evolution of populations [20,21] by allowing for the evolution of local adaptation and the recolonization of new habitat after disturbance [22]. From a proximate perspective (*sensu* [23]), anadromous salmon imprint to the natural odours of natal sites as juveniles in freshwater habitats, migrate to the ocean to feed and grow and migrate homeward as adults using their odour memories [24]. The homing migration occurs only once in the lifetime for semelparous species (i.e. Pacific salmon); all such individuals die within a few weeks of spawning [21]. From an ultimate perspective, natural selection appears to favour the return of locally adapted individuals to their natal sites [25,26], although straying may be adaptive during periods of habitat loss [27] or gain [28]. Rates of homing and straying, therefore, probably reflect a dynamic balance between natural selection pressures specific to each river [18,20,29] and habitat within rivers [19,30,31]. Given that straying permits gene flow among populations [20], straying of genetically distinct hatchery-produced salmon is a conservation concern in many regions, including Alaska [32–34], California [35,36] and the Columbia River basin of the US Pacific Northwest [37–39]. Thus, the study of homing and straying has both fundamental and applied conservation significance.

Straying can be viewed from the perspective of the donor population that *produces* strays, and the recipient population that *receives* strays from donor populations [39–41]. While many studies have estimated the rates of donor or recipient straying, the factors influencing individuals to stray away from donor populations and into recipient populations have received less attention (though see [42] for a review). Evidence suggests a heritable basis to orientation and homing capacity in migrating salmon [43,44], but homing and straying are clearly influenced by anthropogenic factors such as hatchery practices and artificial transportation of smolts [36,38,45–48], biotic factors including the density of conspecifics [42,49,50] and abiotic factors such as temperature [51,52]. It remains unclear to what extent straying reflects an individual failure to home or a choice to stray.

What determines the attractiveness of sites to homing and straying salmon? Given the evidence that strays tend to enter non-natal sites that are close to home waters [32,33,40,41], the distance between donor and recipient populations is often treated as the sole factor determining the distribution of strays (e.g. [53]). However, distance is often insufficient to explain patterns of straying (e.g. [26,40,41,54]; summarized in [21]. Regional and site-specific temperatures appear to influence donor and recipient straying [51,52], and salmon temporarily use non-natal streams as cool-water refuges during upriver migration (e.g. [55,56]), but the physical factors making streams more or less attractive to strays are not clear.

In this article, we combined estimates of donor and recipient rates of straying across 23 spawning seasons of 19 hatchery populations of Chinook salmon, *O. tshawytscha*, to test the hypotheses that the relative attractiveness of rivers is influenced by (i) spatial isolation among rivers, (ii) the size and organization of local rivers in the larger river network, (iii) river temperatures encountered by upstream migrating salmon at confluence junctions and at the natal sites, (iv) the river’s flow during the spawning season, and (v) the density of returning adult salmon. Our corresponding objectives were to: (i) evaluate the consistency of the identified donor and recipient straying patterns for each river through time and (ii) quantify the extent to which these rates are influenced by site-specific characteristics such as watershed size, isolation, streamflow, temperature and salmon density.

## 2. Material and methods

### 2.1. Study site

**Figure 1.**
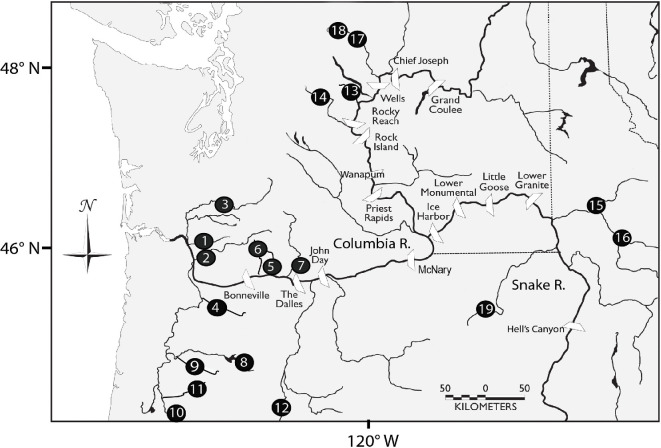
Map of the Columbia River basin showing locations of hatchery populations of Chinook salmon and mainstem dams as white wedges. Numbers correspond to hatchery populations and river names in tables 1 and 2.

We examined patterns of homing and straying in hatchery-produced Chinook salmon populations in the 673 400 km^2^ Columbia River basin, USA (figure 1). For consistency, we limited analyses to releases of individuals consistent with the life history form termed ‘stream-type’, that had spent a full year in the hatchery. This life history type of Chinook salmon tends to stray at lower rates than the ‘ocean-type’ that migrates to sea in its first year of life (e.g. [49], The hatchery programmes in the Columbia River provide a unique, large-scale research opportunity to identify factors associated with homing and straying because, for decades, many millions of juvenile salmon have been marked with coded wire tags (CWTs). CWTs are *ca* 1 mm sections of coded magnetized wire that are inserted into the cranial cartilage of juveniles [57], indicating year and site of release. Recoveries of mature adults in spawning sites and hatcheries allow quantification of rates of homing and straying. In addition to extensive tagging, widespread systematic sampling of wild and hatchery populations throughout the basin and along the coast provides the unprecedented opportunity to detect strays, which is vital as estimates of straying rates are largely influenced by the number of sites examined for tags [47]. Importantly, the tags are read at centralized laboratories, thus the data are ‘blind’ with respect to the collection, eliminating any bias towards recovery of local fish. We included information from hatchery facilities and spawning ground recoveries, representing nearly 450 sites where salmon were sampled. Strays in our dataset were recovered at 164 unique locations, all but 15 within the Columbia River basin. All recoveries of individuals, including at sites outside of the Columbia River basin, were included in estimated rates of donor straying. Given the nearly universal effort to examine all individuals in hatcheries for the presence of CWTs, we are confident in the robustness of the dataset for analysis and interpretation.

**Table 1.** Characteristics of rivers of focal hatchery populations of Chinook salmon in the Columbia River basin, USA used in analysis. Hatchery names correspond to the RMIS database (http://www.rmpc.org/) and are listed in ascending distance (km) upstream from the mouth of the Columbia River (footnote numbers correspond to map in figure 1). Isolation is the water distance (km) from the confluence of each river and the Columbia mainstem to the nearest source of potential foreign strays. An isolation of 1 km indicates that rivers intersect (e.g. Marion Forks Hatch on the North Santiam River intersects with the South Santiam River to form the mainstem Santiam). Area is watershed size of river sub-basins (km^2^), streamflow is observed August discharge (m^3^ s^-1^), local river water temperature (°C) in August, confluence temperature (°C) is the temperature at the first major downstream junction from each river in the stream network. Escapement is the total estimate of salmon returning to hatcheries (local+foreign) as estimated from CWT expansions. Values are averages ± 1 s.d.

water temperature (^o^C)								
**hatchery population**	**river name**	**area**	**isolation**	**distance**	**streamflow**	**local**	**confluence**	**escapement**
Fallert Cr. Hatchery^1^	Kalama	463	11	97	57.1 ± 21.2	16.1 ± 0.4	22.0 ± 0.4	506 ± 544
Lewis River Hatchery^2^	Lewis	1245	18	121	40.9 ± 11.0	14.3 ± 0.4	16.6 ± 0.4	406 ± 344
Cowlitz Salmon Hatch^3^	Cowlitz	6423	11	136	97.4 ± 23.3	15.4 ± 0.4	21.7 ± 0.4	2206 ± 1926
Clackamas Hatchery^4^	Clackamas	2434	137	186	25.6 ± 3.9	16.9 ± 0.4	19.2 ± 0.4	3822 ± 1696
Little White Salmon NFH^5^	Ltl. White Salmon	347	14	229	6.7 ± 2.3	13.4 ± 0.5	20.6 ± 0.5	6062 ± 2354
Carson NFH^6^	Wind	582	14	235	6.8 ± 1.3	11.1 ± 0.4	14.9 ± 0.4	2675 ± 2360
Klickitat Hatchery^7^	Klickitat	3359	28	306	26.4 ± 7.4	12.4 ± 0.6	21.7 ± 0.6	332 ± 264
South Santiam Hatchery^8^	Santiam	1657	1	346	21.7 ± 3.2	13.0 ± 0.4	18.7 ± 0.4	1930 ± 1121
Marion Forks Hatchery^9^	Santiam	1781	1	386	28.6 ± 3.1	12.4 ± 0.4	18.6 ± 0.4	1561 ± 778
Willamette Hatchery^10^	MF Willamette	5257	1	403	81.2 ± 11.8	11.2 ± 0.4	17.9 ± 0.4	2143 ± 1372
McKenzie Hatchery^11^	McKenzie	3462	1	409	37.5 ± 11.6	13.8 ± 0.4	17.9 ± 0.4	2543 ± 1612
Round Butte Hatchery^12^	Deschutes	27 195	15	433	111.8 ± 8.9	12.1 ± 0.4	19.3 ± 0.4	578 ± 240
Entiat NFH^13^	Entiat	1085	63	715	6.4 ± 3.5	16.4 ± 0.7	17.3 ± 0.7	670 ± 615
Leavenworth Hatchery^14^	Wenatchee	3369	24	718	35.7 ± 23.5	14.9 ± 0.7	18.4 ± 0.7	2931 ± 1891
Dworshak Nat. Hatchery^15^	Clearwater	24 967	52	725	353.2 ± 97.8	8.4 ± 0.6	14.9 ± 0.6	1540 ± 809
Kooskia NFH^16^	MF Clearwater	3489	52	775	57.8 ± 19.2	21.7 ± 0.6	17.5 ± 0.6	599 ± 383
Winthrop NFH^17^	Methow	4687	63	823	12.6 ± 5.6	13.5 ± 0.5	18.4 ± 0.5	824 ± 873
Methow Hatchery^18^	Methow	4687	63	825	11.8 ± 5.5	19.8 ± 0.6	18.4 ± 0.6	246 ± 208
Lookingglass Hatchery^19^	Grand Ronde	3626	100	1282	18.1 ± 6.6	11.1 ± 0.6	21.8 ± 0.6	245 ± 342

**Table 2.** Tag recoveries and donor and recipient rates of straying for hatchery populations of Chinook salmon in the Columbia River basin, USA during return years 1993−2016. Hatchery names correspond to the RMIS database (http://www.rmpc.org/) and are listed following table 1. Tagged fish were categorized as home if they were recovered in their river of release, stray if they were recovered in a river they had never been reared in or released in, and ambiguous if recovered at a river where they had been reared but not released. The average percentage of straying reflects the *annual* estimate of donor or recipient straying rate for each population across 23 years of return (i.e. the average is not simply the pooled total number of strays divided by total recoveries).

donor rate									recipient rate				
					**percentage stray**						**percentage stray**		
**hatchery population**	**home**	**stray**	**ambiguous**	**total recoveries**	**average**	**s.d.**	**home**	**stray**	**ambiguous**	**total recoveries**	**average**	**s.d.**	**distance**
Fallert Cr. Hatchery^1^	10 663	546	0	11 209	5.3	6.0	8260	342	0	8602	9.03	15.7	97
Lewis River Hatchery^2^	14 581	271	33	14 885	2.5	2.5	4190	103	23	4316	0.21	0.2	121
Cowlitz Salmon Hatch^3^	61 753	974	162	62 889	0.2	0.4	50 579	733	152	51 464	4.31	4.4	136
Clackamas Hatchery^4^	108 512	4578	501	113 591	1.0	1.7	86 633	1441	307	88 381	0.66	0.7	186
Little White Salmon NFH^5^	230 888	15 998	21	246 907	2.5	2.8	117 268	22 161	20	139 449	17.20	12.2	229
Carson NFH^6^	78 232	13 911	18	92 161	17.3	15.2	58 725	131	9	58 865	0.31	0.7	235
Klickitat Hatchery^7^	26 370	598	0	26 968	7.8	25.6	7484	74	0	7558	1.13	2.5	306
South Santiam Hatchery^8^	51 499	1781	552	53 832	7.8	11.9	44 277	112	221	44 610	0.20	0.4	346
Marion Forks Hatchery^9^	46 821	1854	330	49 005	1.7	4.0	24 471	104	323	24 898	6.06	13.3	386
Willamette Hatchery^10^	60 735	13 142	18	73 895	8.8	14.3	51 342	363	11	51 716	2.50	5.7	403
McKenzie Hatchery^11^	71 998	2088	62	74 148	1.2	1.2	55 514	3019	62	58 595	6.04	13.0	409
Round Butte Hatchery^12^	26 868	87	425	27 380	0.6	1.4	7009	304	43	7356	5.36	8.5	433
Entiat NFH^13^	15 296	569	0	15 865	5.7	14.7	13 150	1182	0	14 332	1.64	2.4	715
Leavenworth Hatchery^14^	79 972	2012	9	81 993	0.9	1.1	67 014	408	2	67 424	0.35	0.5	718
Dworshak Nat. Hatchery^15^	38 788	2910	500	42 198	16.9	31.4	31 350	994	457	32 801	4.22	5.6	725
Kooskia NFH^16^	15 854	2354	58	18 266	24.5	36.3	11 904	666	28	12 598	5.36	7.3	775
Winthrop NFH^17^	16 441	798	35	17 274	8.6	22.2	17 255	50	33	17 338	3.57	12.9	823
Methow Hatchery^18^	9147	730	2	9879	0.8	0.9	3696	6	2	3704	2.10	7.1	825
Lookingglass Hatchery^19^	30 620	3401	25	34 046	5.7	5.7	1979	18	2	1999	0.82	1.6	828
**GRAND TOTALS**	**995 039**	**68 602**	**2751**	**1 066 392**			**662 100**	**32 211**	**1695**	**696 006**			

### 2.2. Considerations of straying nomenclature

Analyses of homing and straying are sensitive to issues of scale [31,39,58], thus clarification of terminology is essential. We quantified homing and straying to the sub-basin scale corresponding to individual river systems, consistent with many previous studies in the basin [41,45,49,52,59,60]. Each hatchery population was assigned to the river system within the Columbia River watershed where the fish were released and were assumed to imprint and return if they homed (see details below). Despite the inevitable complexities inherent in the diverse size and configuration of the rivers in this huge basin, this spatial scale is largely synonymous with designations of ‘populations’ as conservation units [61] and consistent with other analysis of population connectivity in the basin [60]. For interpretability, we hereafter refer to sub-basins within the river network simply as rivers. CWTs were recovered from carcasses during spawning surveys or when fish were euthanized for spawning at a hatchery, and we assumed that fish recovered in rivers or hatchery collection facilities entered those areas volitionally to reproduce.

### 2.3. Operational definition of home and stray

Tagged salmon were categorized as having ‘homed’ if they were recovered in the river of release, or ‘strayed’ if recovered in another river. Some hatchery-produced fish were reared in one location and released elsewhere, we categorized recoveries as ‘ambiguous’ if the fish returned to sites experienced during incubation or rearing but that were not the river where they were released. Displacing fish from one location to another can influence straying [47], but a complementary analysis of donor straying rates using a shorter-term dataset of our focal populations revealed no clear influence of such displacement at this spatial scale of analysis [52], so we did not include that factor here. In rivers with more than one hatchery (e.g. Kalama Falls Hatchery and Fallert Creek Hatchery are both on the Kalama River), fish released from one hatchery but recovered at another hatchery in the same river were considered to have ‘homed’. Additionally, we excluded releases what were flagged as ‘experimental’ in the Regional Mark Information System (RMIS database, as these fish may have experienced conditions during rearing or release that may confound our interpretation.

### 2.4. Estimating stray rates

We updated an existing dataset used by Westley *et al.* [52], compiled from the RMIS (available at: http://www.rmpc.org/) maintained by the Regional Mark Processing Center of the Pacific States Marine Fisheries Commission. These populations were originally chosen as they provided geographical coverage throughout the basin and had long periods of largely uninterrupted data collection, which we have updated for the return years 1993−2016.

Prior to analysis, each recovery was subjected to a two-step process to account for (i) the fraction of a sample of fish examined for tags and (ii) the proportion of released fish that were tagged [62]. By doing so, we estimated the absolute number of fish represented by each tag recovery. The median expansion factor resulting from sampling fractions was 1.01, and the median ratio of untagged to tagged fish was 3.03. Thus, each recovery in our dataset represented on average *ca* 3 total fish. Our analyses excluded fish caught in the ocean or in rivers en route to spawning areas because they did not have the opportunity to home or stray, and we made no effort to account for natural mortality or mortality from fisheries.

Donor rate for each of the 19 focal hatcheries in each year was calculated by dividing the total number of fish released from that hatchery programme that was determined to have strayed divided by the sum of the fish from that programme that homed and strayed. Recipient rate for each of the 19 focal hatcheries in each year was calculated by dividing the total number of strays recovered in that hatchery by the sum of the local fish and strays. Thus, while the donor and recipient rates are related, they are not entirely contingent on the other given distinctly different denominators. But to be clear, donor rates of straying included recoveries from all sites, not just focal locations, and recipient rates included recoveries of strays from any hatchery programme not limited to only the 19 focal hatcheries, though strays certainly occur among the focal sites.

### 2.5. Habitat covariates

We compiled the following river features (table 1) corresponding to the river of each hatchery population:

(1) A proxy for ‘patch size’: drainage area of each river’s sub-basin was downloaded from: http://waterwatch.usgs.gov/ and converted from square miles to km^2^
(2) A proxy for ‘patch isolation’: water distance (km) from the most downstream end of each population’s river boundary to the nearest river boundary of potential sources of strays was estimated using the path tool in Google Earth Pro 7.1.2.2041.
(3) A proxy for migration distance: the minimum distance (km) a fish must swim from the mouth of the Columbia River at the Astoria Bridge to the geographical coordinates of each hatchery using the path tool in Google Earth Pro 7.1.2.2041. This value provided an additional measure of isolation as rivers closer to the Columbia River’s mouth experience more potential strays swimming past than do rivers farther upstream.
(4) Proxies for ‘patch quality’: average August water temperatures (°C) during 1993−2016 were extracted from the NorWeST project database (http://www.fs.fed.us/rm/boise/AWAE/projects/NorWeST.html, Dan Isaak, personal communication). Estimated water temperatures are derived from spatial-statistical stream network models fitted to observed water temperature data of more than 15 000 stream sites [63,64]. This model provides both predictive accuracy (*r*^2^ = 0.91; RMSE = 1.0°C) and an unbiased approach to assessing water temperatures across large geographical regions. The thermal conditions in August highly correlate with conditions in other summer months [65] and can thus be used as a proxy for summer water temperature at sites within a given spawning season. Water temperature referred to here as local temperature was estimated at sites immediately adjacent to geographic coordinates of each hatchery location (as downloaded from http://www.rmpc.org/) and (i) at the last major downstream confluence junction from the hatchery location that individuals of that population would encounter while migrating upstream, and (ii) used differences between local and confluence (‘diff’ temperature) to explore the relative differences between temperatures experienced en route versus at hatcheries. For example, at the Dworshak hatchery location, we assessed ‘local’ temperature adjacent to the fish ladder in the branch of the Clearwater River draining Dworshak Reservoir, and ‘confluence’ temperature approximately 700 m downstream at the junction where upstream migrating fish would be faced with a decision of turning left to ascend towards the hatchery or go right and continue up the mainstem Clearwater River. In addition to temperature, the average streamflow in the month of August was retrieved for each hatchery given its location in the river network from http://waterwatch.usgs.gov/ and used as an additional proxy for stream quality.
(5) A proxy for density dependence and potential social interactions [66] among upstream migrating fish; we used the total number of adult fish sampled in each hatchery in each year as a measure of spawner density. We hypothesized that large returns of local fish would attract strays into non-natal rivers, perhaps by the mechanism of socially influenced collective navigation [50].

### 2.6. Statistical model

**Figure 2.**
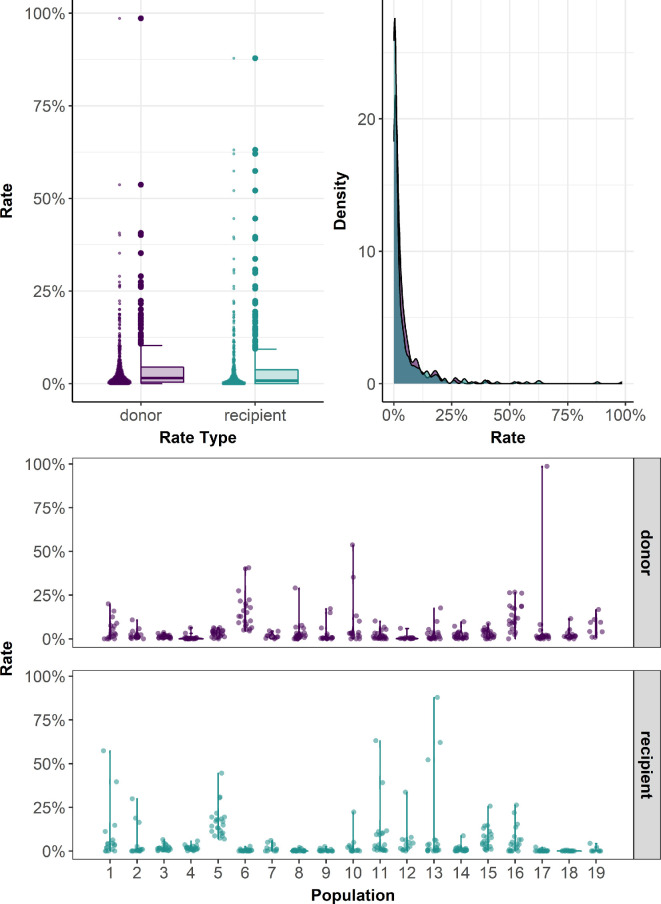
Observed donor (purple) and recipient (blue) rates from 19 hatchery populations of Chinook salmon in the Columbia River basin. The top left panel shows violin (left) and box-and-whisker (right) plots of the observed distributions of donor and recipient rates aggregated across all 19 populations. The top right panel shows the kernel density plots of both rates. The bottom panel shows violin plots of observed donor and recipient rates for each population.


The donor and recipient rates derived from the previously described methods are non-negative continuous data between zero and one, with inflation at zero and (potentially) one. A considerable proportion of these observations were zero values (figure 2), and the data are right-skewed and heteroscedastic [67]. Therefore, we assumed the observed donor and recipient rates could be modelled with a beta distribution with inflation at zero and/or one. We assumed $Y_{j}$ is the response variable (the donor or recipient rate) measured on n experimental groups/populations $Y_{j}=\left(Y_{1}\ldots Y_{n}\right)$.

$$Y_{j}=\left(Y_{donor,\;j},Y_{recipient,\;j}\right).$$

It was assumed that $Y_{j}$ follows a piecewise distribution when $Y_{j}$ has inflation at zero and/or one,


(2.1) $$f(Y_{j})=\left\{ \begin{array}{ll} \;\;\;p_{j}, & Y_{j}=0\\ \;\;(1-p_{j})q_{j}, & Y_{j}=1\\ (1-p_{j})(1-q_{j})\;Beta(\alpha_{j1},\alpha ), & Y_{j}\in (0,1) \end{array}\right.$$

where $p_{j}$ is the probability that $Y_{j}=0$, $q_{j}$ is the probability that $Y_{j}=1|Y_{j}\neq 0$ and $\alpha_{j1}$ and $\alpha_{j2}$ are the two parameters of the beta distribution when $Y_{j}\in \left(0,1\right)$. The parameters from the beta and binomial distributions are linked to the design matrix of the model’s explanatory variables and the latent variables through the following link functions:


(2.2) $$logit\left[\mu_{j}^{\left(0,1\right)}\right]=\beta_{0,k}+\beta_{1,k}x_{j}+b_{0,k,g[j]}+b_{1,k,g[j]}x_{j}+\epsilon_{k,j}$$

(2.3) $$log\left[\phi \right]=\beta_{0,k}$$

(2.4) $$logit\left[p_{j}\right]=\gamma_{0,k}+\gamma_{1,k}x_{j}+u_{0,k,g[j]}+u_{1,k,g[j]}x_{j}$$

where $\mu_{j}^{\left(0,1\right)}$ is the mean of the beta distribution: $\mu_{j}^{\left(0,1\right)}={\alpha_{j1}\left(\alpha_{j1}+\alpha_{j2}\right)}^{-1}$; ϕ is a dispersion parameter that captures the variation of the beta distribution ($\phi =\alpha_{j1}+\alpha_{j2}$), which is assumed to be constant across all experimental groups/populations. This is relevant because it allows calculation of the beta distribution’s variance: $Var\left[Y_{j}|Y_{j}\in \left(0,1\right)\right]=\mu_{j}^{\left(0,1\right)}\left[1-\mu_{j}^{\left(0,1\right)}\right]={\left(\phi +1\right)}^{-1}$. We evaluated preliminary model formulations wherein ϕ was allowed to vary by population, but this led to poorer model convergence and did not result in any meaningful improvement to the model fit, according to the information criterion (discussed later in this methods section).

k is the response type (donor or recipient); $\beta_{0,k}$ and $\beta_{1,k}$ are the fixed-effect coefficients; $b_{0,k,g[j]}$ and $b_{1,k,g[j]}x_{j}$ are the random intercepts and slopes for population $g\left[j\right]$, and $\epsilon_{k,j}$ is the term representing the error residuals. $\gamma_{0,k}$ and $\gamma_{1,k}$ are the fixed-effects coefficients, and $u_{0,k,g[j]}$ and $u_{1,k,g[j]}$ are the random intercepts and slopes for population $g\left[j\right]$ in the zero-inflation model. Finally, because there were no observed donor or recipient rates that were equal to one, we did not estimate $q_{j}$ and, thus, did not include its link function above. Non-informative priors were imposed on all estimated model coefficients, and all independent variables were centred by subtracting the mean and standardized by dividing by their standard deviations. Random effects are specified for both the intercepts and slopes for equations (2.2) and (2.4). For example, in equation (2.2), they are modelled as: $\beta_{0,k,g}\sim N\left(0,\sigma_{0,k}^{2}\right)$and$\beta_{1,k,g}\sim N\left(0,\sigma_{1,k}^{2}\right)$, where $\sigma_{0,k}^{2}$ and $\sigma_{1,k}^{2}$ are the variances of the random intercept and slope for the response k.

Parameter estimation was conducted via Bayesian inference with the Stan modelling software, which was implemented with the ‘brms’ package [68] in the R programming environment [69]. For each model, we generated six Markov chain Monte Carlo (MCMC) chains, each containing 4000 samples, discarded the first half of each chain, then used the remaining 2000 samples in each chain to infer the marginal posterior distribution of each model parameter. We assessed convergence of the MCMC chains by visually inspecting trace plots of all parameters and calculation of the Gelman–Rubin diagnostic statistics [70]. Convergence of the MCMC chains was assumed if Gelman–Rubin statistics were less than 1.01, and there were no patterns in the trace plots that would suggest autocorrelation in the MCMC chains [71]. The ability of each candidate model to reproduce the observed data was evaluated using leave-one-out cross-validation, using the ‘loo’ package [72].

## 3. Results

### 3.1. Donor and recipient rates of straying

**Figure 3.**
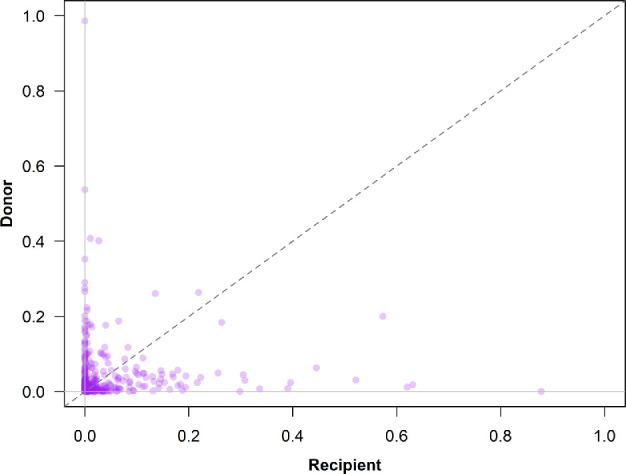
Bivariate plot of observed donor (y-axis) and recipient (x-axis) rates for 19 populations of hatchery-origin Chinook salmon across 23 years of return to the Columbia River basin. The dashed diagonal grey line depicts the 1 : 1 line through the plot origin.

A total of 358 river-year paired comparisons of donor and recipient stray rates of Chinook salmon were available from 19 focal rivers across 23 spawning seasons. These sources produced recovery data on 1 066 394 salmon for assessment of donor straying patterns and 616 507 salmon for assessment of recipient straying patterns (table 2). The total numbers of recoveries differed as all individuals recovered from any location were included in estimates of donor straying, whereas only recoveries in the subset of focal hatcheries were included for recipient rates. The average annual donor rate ranged from 0.6 to 15.3% among populations, and the average recipient straying rate ranged from 0.07 to 16.7% (figures 2 and 3 and table 2), indicating that the rates of straying are generally small but highly variable among populations and years.

### 3.2. Influence of river features on patterns of recipient and donor straying

Models that included all habitat covariates performed better than those that omitted some and drastically outperformed univariate models (electronic supplementary material, table 4. However, our model selection criteria implied that there was *not* a meaningful difference in predicative ability between the full model that included water temperature at the tributary confluence and one that included local water temperature (electronic supplementary material, table 4). Therefore, we included the results for both models in our discussion of the most important habitat covariates (table 3). The two best-performing models did not include annual year effects but did include random effects for covariates that varied on an annual basis, like water temperature (confluence and local), escapement and flow (electronic supplementary material, table 4). Covariates that were stationary (e.g. basin area, distance, isolation) were treated as fixed effects in all candidate models.

**Table 3.** Model coefficients from the two best-performing candidate models for predicting donorship and straying rates of hatchery-origin Chinook salmon in the Columbia River basin. Coefficients associated with the mean (*µ*) of the zero/one inflated beta (ZOIB) model are shown on the top half of the table, while the coefficients associated with $P\left(Y=0\right)$(p) are listed on the bottom half. The medians and the 95% credible intervals of the marginal posterior distributions for each coefficient are shown. Covariates that do not overlap with zero are shown in bold print. For explanation of parameter values, see table 1

		*µ*					
		*donor*			*recipient*		
model	parameter	median	2.5%	97.5%	median	2.5%	97.5%
1	area	−0.41	−0.91	0.07	−0.25	−0.69	0.20
	distance source	−0.11	−0.47	0.26	−0.02	−0.34	0.32
	distance	0.12	−0.34	0.58	−0.05	−0.47	0.35
	local flow	0.15	−0.42	0.77	0.40	−0.13	0.94
	**escapement**	**−0.40**	**−0.68**	**−0.11**	**−0.43**	**−0.70**	**−0.16**
	diff. temp.	−0.08	−0.60	0.49	−0.04	−0.51	0.45
	confluence temp.	0.15	−0.28	0.57	0.06	−0.42	0.44
2	**area**	**−0.49**	**−0.96**	**−0.02**	−0.29	−0.74	0.17
	distance source	−0.07	−0.42	0.29	−0.04	−0.39	0.32
	distance	0.13	−0.29	0.54	0.04	−0.35	0.42
	local flow	0.11	−0.45	0.66	0.39	−0.16	0.94
	**escapement**	**−0.39**	**−0.68**	**−0.11**	**−0.42**	**−0.69**	**−0.14**
	diff. temp.	0.18	−0.41	0.77	0.13	−0.46	0.70
	local temp.	0.19	−0.40	0.80	0.16	−0.35	0.64
		*p*					
		*donor*			*recipient*		
model	parameter	median	2.5%	97.5%	median	2.5%	97.5%
1	area	0.58	−1.13	2.47	−0.44	−2.70	1.64
	distance source	−0.28	−1.36	0.79	−0.30	−1.67	1.09
	distance	−0.64	−2.17	0.82	0.88	−0.89	2.84
	local flow	−1.44	−3.95	0.93	−2.57	−5.57	0.01
	**escapement**	−0.37	−1.59	0.80	**−1.23**	**−2.04**	**−0.55**
	diff. temp.	−0.25	−2.07	1.33	1.14	−0.62	3.23
	**confluence temp**.	−0.89	−2.63	0.59	**−1.32**	**−2.84**	**−0.05**
2	area	0.34	−1.40	2.36	−0.41	−2.53	1.56
	distance source	−0.44	−1.68	0.76	−0.26	−1.64	1.09
	distance	−0.30	−1.88	1.09	0.72	−0.91	2.60
	local flow	−0.76	−3.13	1.75	−2.45	−5.27	0.09
	**escapement**	−0.42	−1.61	0.75	**−1.23**	**−2.06**	**−0.56**
	diff. temp.	−1.45	−4.01	0.56	−0.78	−3.00	1.28
	**local temp**.	−0.71	−2.80	1.19	**−1.77**	**−3.71**	**−0.06**

**Figure 4.**
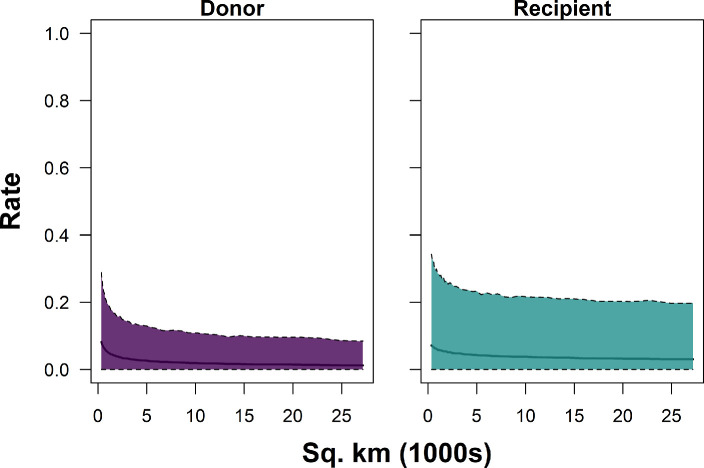
Predicted relationship between watershed/basin size (area; 1000 km^2^) and donor (left) and recipient (right) rates of Chinook salmon in the Columbia River, for unsampled tributaries, based on one of the two best-performing models (Model 2).

**Figure 5.**
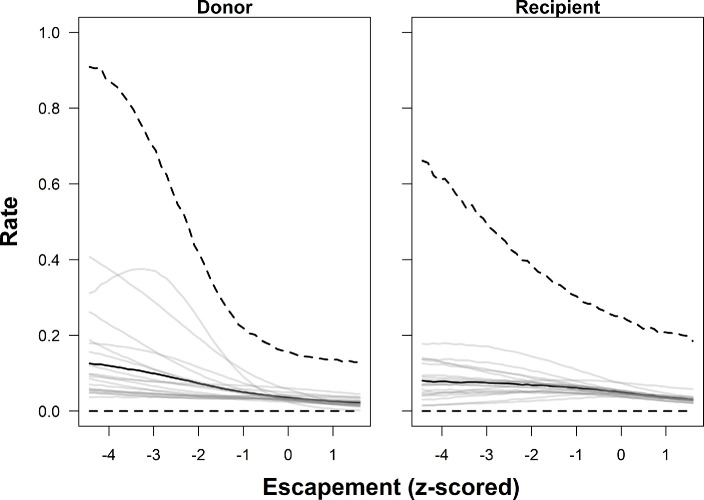
Predicted relationships between escapement (standardized) and donor (left) and recipient (right) rates of Chinook salmon in the Columbia River, based on the estimated parameters of Model 1. The thick black line depicts the posterior predictive distribution (i.e. an unsampled population) median, while the thick dashed lines show the 95% credible intervals. The thin grey lines are the posterior medians of each of the 19 populations included in the study.

**Figure 6.**
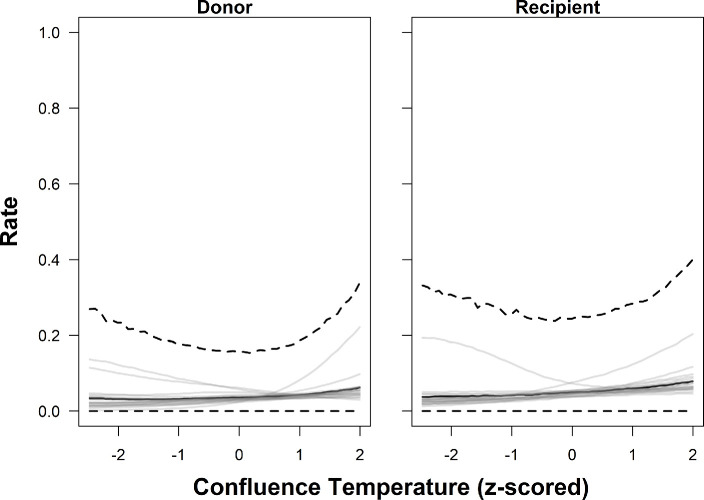
Predicted relationships between confluence temperature (standardized) and donor (left) and recipient (right) rates of Chinook salmon in the Columbia River, based on the estimated parameters of Model 1. The thick black line depicts the posterior predictive distribution (i.e. an unsampled population) median, while the thick dashed lines show the 95% credible intervals. Thin grey lines are the posterior medians of the 19 individual populations included in the study.

**Figure 7.**
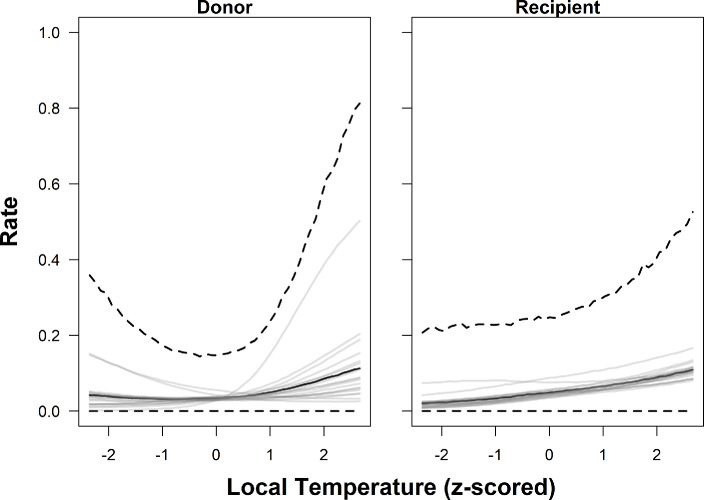
Predicted relationships between local temperature (standardized) and donor (left) and recipient (right) rates of Chinook salmon in the Columbia River, based on the estimated parameters of Model 2. The thick black line depicts the posterior predictive distribution (i.e. an unsampled population) median, while the thick dashed lines show the 95% credible intervals. Thin grey lines are the posterior medians of the 19 individual populations included in the study.

The two best-performing models indicated that basin area, salmon abundance, and confluence and local water temperature were most strongly associated with observed donor and recipient rates (i.e. the marginal posterior distribution did not overlap with zero) (table 3; figures 4–7). Greater basin area was strongly associated with lower rates of donor straying but not recipient rates. Thus, proportionally fewer salmon strayed from larger basins than from smaller basins (table 3; figures 4, electronic supplementary material, figures 10–12). Similarly, donor and recipient rates both decreased with increasing salmon abundance (table 3; figure 5). Thus, when more salmon from a given river survived to return, a higher proportion of them homed compared with years when fewer returned, and a lower proportion of salmon from other rivers entered that river as strays.

Both donor and recipient rates of straying were positively associated with temperature-related covariates (confluence and local temperature, table 3). That is, when the natal stream was warm relative to the river the salmon were ascending to reach it, and when it was warm at the designated local home site, larger proportions of the population strayed elsewhere. However, the probability of the recipient rates being equal to zero had a strong negative association with confluence and local temperature, which suggests that cooler temperatures increase the chance of a tributary receiving *some* strays. It is worth noting there was some among-population variability, with certain populations having the opposite relationship, relative to the average/global trend (figures 6,7). For example, both Carson National Fish Hatchery (Wind River, Washington) and Willamette (Willamette River, Oregon) hatcheries had decreasing donor rates with increasing stream temperature (local and confluence) (electronic supplementary material, figures 10 and 11).

## 4. Discussion

Why are certain rivers attractive or unattractive to homing and straying salmon? Our analyses yielded several salient insights into this complex, unresolved question. First, over half the combinations of years and sites had low rates of straying (less than *ca* 2%) into and from these sites. This result is consistent with previous observations that straying rates are typically low for Chinook salmon with stream-type life history (i.e. they spend a year in fresh water prior to seaward migration and return in the spring and early summer [49]). Second, rivers differed in long-term average rates of donor (range 1–10%) and recipient (3–13%) straying, indicating the importance of features that are either fixed (e.g. location and basin area) or annually varying but nevertheless inherent features of the river (e.g. water temperature and discharge). Third, water temperature, both in tributary rivers associated with focal hatcheries and at confluences downstream of hatcheries, significantly influenced straying. As temperatures rose, both donor and recipient rates of straying tended to increase, which suggests that warming temperatures are likely to impact both the numbers of fish leaving and entering sites. Fourth, abundance of local fish was negatively associated with increased probabilities of both donor and recipient straying, consistent with social behavioural effects of collectively migrating individuals. Fifth, we did not detect significant effects of distance of tributary upstream from the ocean, or distance to nearest source of strays, suggesting that simple predictions of patch dynamics fail to describe the dynamics of this system. Taken together, these results support the mounting evidence that factors associated with site quality, beyond site isolation, must be considered in analyses of metapopulation dynamics.

Consistent with previous findings on the geographical patterns of straying in Chinook salmon [40,41,49,59], we observed that certain rivers tended to *produce* strays and others tended to *attract* them. This suggests that a suite of generally stable factors, potentially both biological and abiological, act within rivers to shape patterns of homing and straying. This is perhaps not surprising given that temporal stability in the ‘bouquet’ of odorants used in imprinting and subsequent upstream migration by adults is a requisite characteristic of the olfactory hypothesis [73]. Indeed, sites tended to be consistent in their propensity to produce or attract strays. For example, the Carson Hatchery population site consistently produced high rates of straying, whereas the Clackamas and Round Butte sites consistently produced few strays. The most consistently attractive site, Little White Salmon, averaged 16.7% recipient stray rates; in contrast, the geographically proximate Carson Hatchery averaged 0.6% underscoring variable attractiveness across the landscape. Importantly, however, both donor and recipient rates of straying were highly variable and 95% credible intervals at every site for both measures of straying were bounded by zero. This site-specific year-to-year variation reflects the dynamic habitats in which Pacific salmon have evolved [74] and is consistent with a waxing and waning of site suitability across decades.

Water temperatures at both local rivers and at river confluences downstream of local rivers that would be encountered by upstream migrating salmon were positively associated with donor and recipient rates of straying. This is consistent with observed increased donor rates of straying by migrating Chinook salmon in years of relatively warm water in the mainstem Columbia River [52]. Similarly, Snake River fall returning Chinook salmon were more likely to stray into the Upper Columbia River when the Snake River itself was warm [48]. We interpret the increase of recipient straying in years of relatively warm conditions to be consistent with the observations of upstream migrants temporarily entering cool non-natal rivers to avoid high temperatures in mainstream corridors [48,51]. As water temperatures rise in the face of a warming global climate system, maintaining connectivity and options for mobile, cold-water-adapted species is likely to be increasingly important targets for conservation [75]

We detected negative associations between local conspecific abundance (measured as the number of local fish returning to hatchery sites) with both donor and recipient rates of straying. That is, when the local population was more abundant, higher proportions of those fish homed to that river, and lower proportions of strays were detected entering. These findings corroborate results of donor rates of straying reported by others [52,59,76] and are generally consistent with predictions of social interactions leading to emergent properties of collective navigation [50]. Moreover, social interactions appear to help Chinook salmon during the upstream migration to navigate the difficult conditions around mainstem dams [66]. A negative association between recipient rates of straying and conspecific abundance has recently been described for upper Columbia River salmon [77], and our data also suggests that strays may tend to go elsewhere when local conspecific densities are high. This is not consistent with a pied piper effect where large numbers of local individuals may lead non-local hatchery fish onto the spawning grounds. However, it is consistent with the ability of salmon to distinguish the odours of their population from other populations (e.g. [78]). These social or chemical factors associated with the local population may have reduced the tendency of salmon to enter when local salmon were very abundant, though these pheromones play a secondary role to that of imprinted odours in the overall homing process [79]. Regardless of the mechanism, this result underscores the importance of maintaining local spawning population sizes, which should lower the chances of the population being overwhelmed by hatchery strays that might reach the spawning grounds [42].

Although the spatial isolation or distance among sites is frequently used as a proxy for likelihood of population connectivity, we found no evidence that sites closer to potential sources of strays were more likely to have higher recipient rates of straying. Previous work tends to show that, all else being equal, hatchery strays use sites that are closer to locations from which they were released: for example, pink salmon (*O. gorbuscha*) and chum salmon (*O. keta*) populations in Alaska [32] and Chinook salmon in the Sacramento River basin [80]. However, our results indicate that salmon population connections reflect more than spatial isolation. This finding is consistent with work on other populations of Chinook salmon in the Columbia River [41] and Chinook salmon established outside their native range in New Zealand [54]. Both of those studies reported many exceptions to the overall relationship between spatial proximity and straying.

Our work not only confirms that site-specific factors that are fixed among years and ones that vary are associated with the homing and straying of Pacific salmon but has implications for the functioning of metapopulation dynamics. Indeed, Fleishman *et al.* [11] similarly showed that habitat quality (e.g. disturbance frequency and presence of host plants) superseded the role of patch size or isolation in butterfly population dynamics. Similarly, a sockeye salmon (*O. nerka*) metapopulation in the Fraser River, Canada, was greatly influenced by the connectivity of individuals dispersing between sites of consistently high habitat quality to sites of lower quality, especially in years of high salmon abundance [81]. Beyond site-specific factors that are largely static in time, recent evidence points to changing weather and climatic effects as having effects on metapopulation dynamics. For example, dynamics of a butterfly (*Melitaea cinxia*) metapopulation were shown to parallel the synchrony of spring precipitation, with climate change increasing metapopulation synchrony and associated extinction risk [82]. Our results pointed strongly to a role of increasing water temperatures to increase dispersal rates both into and out of local populations. This suggests that warming may put added stress on many already depleted salmon populations by stimulating dispersal away from local sites while also appearing to encourage the movement into those sites by fish produced in other rivers. Given that small populations can be easily overwhelmed by non-local fish on the spawning grounds [42], it may be increasingly important to consider the risks of increasing temperature at the metapopulation-level beyond its direct effect on individual physiology or behaviour. Beyond the implications of our work for salmon and other philopatric fishes that must balance homing with avoidance of unfavourable environmental conditions, other species also make trade-offs during migration between chemosensory stimuli and environmental conditions. For example, adult sea lamprey migrate upriver to spawn but do not return to natal sites. Rather, they are attracted to the chemical traces of larval conspecifics and to sexually mature adults [83], but avoid alarm substances from dead adult conspecifics [84]. These complex chemosensory responses are also mediated by water temperature and depth during migration. Similarly, upstream migrating juvenile galaxiid fishes are attracted to the chemical traces of adult conspecifics [85], and this attraction mediates their avoidance of suspended sediments [86].

As human activities and infrastructure such as dams, diversions and land-use practices continues to alter thermal regimes of rivers [87,88], we should expect complex changes in the attractiveness of sites to dispersing individuals with profound repercussions for recipient and donor populations. Water temperatures in the Columbia Basin and Pacific Northwest have warmed substantially in the twentieth century [63,65] and are predicted to continue to warm [89], which will probably influence the phenology of species [90,91] and have other evolutionary effects on populations [92]. Given the limits for adaptation to thermal regimes [93,94], increased rates of straying in Chinook salmon in response to increased temperatures may influence their persistence in a warming world but also alter the trajectories of local populations.

## Data Availability

Data is available at: https://github.com/benjaminnelson/columbia-chinook-straying [95]. Supplementary material is available online [96].
